# Relationship between cognition and frailty in elderly: A systematic review

**DOI:** 10.1590/1980-57642015DN92000005

**Published:** 2015

**Authors:** Allan Gustavo Brigola, Estefani Serafim Rossetti, Bruna Rodrigues dos Santos, Anita Liberalesso Neri, Marisa Silvana Zazzetta, Keika Inouye, Sofia Cristina Iost Pavarini

**Affiliations:** 1 Bacharel, Mestrando do Programa de Pós Graduação em Enfermagem da Universidade Federal de São Carlos; 2 Graduando em Gerontologia da Universidade Federal de São Carlos; 3 Professor Doutor, Docente do Programa de Pós Graduação em Gerontologia da Faculdade de Ciências Médicas da Universidade Estadual de Campinas; 4 Professor Doutor, Docente do Departamento de Gerontologia da Universidade Federal de São Carlos.

**Keywords:** cognition, frail elderly, elderly health, dementia

## Abstract

**Objective:**

The aim of this study was to analyze the relationship between cognition and frailty in the elderly.

**Methods:**

A systematic review on the currently existing literature concerning the subject was carried out. The search strategy included LILACS, SCOPUS, SciELO, PsycINFO, PubMed and Web of Science databases.

**Results:**

A total of 19 studies were selected for review, from which 10 (52.6%) were cross-sectional and 9 (47.4%) longitudinal, and the majority Brazilian. All of the studies established a link between cognition and frailty. There was a relationship between components of frailty and the cognitive domains. Risk of Mild Cognitive Impairment (MCI), dementia and mortality were all evidenced in the relationship between frailty and cognitive impairment.

**Conclusion:**

The theory remains limited, but results show the variables that appear to be linked to cognition and frailty in elderly. This data can help in implementing actions to improve the quality of life among elderly.

## Abstract (pt)

**Objetivo:**

Este estudo objetivou analisar a relação entre cognição e fragilidade em pessoas idosas.

**Métodos:**

Realizou-se uma revisão sistemática da literatura sobre o assunto. A estratégia de busca incluiu as bases LILACS, SCOPUS, SciELO, PsycINFO, PubMed e Web of Science.

**Resultados:**

Foram selecionados 19 estudos para revisão. Dos estudos, 10 (52,6%) eram transversais e 9 (47,4%) longitudinais, sendo a maioria brasileiros. Todos os estudos estabeleceram relação entre cognição e fragilidade. Componentes da fragilidade e os domínios cognitivos apresentaram-se relacionados. Risco de Comprometimento Cognitivo Leve (CCL), demência e mortalidade foi evidenciado a partir da relação entre fragilidade e alterações cognitivas.

**Conclusão:**

A literatura continua limitada, mas os resultados mostram as variáveis que parecem estar relacionadas à cognição e fragilidade em pessoas idosas. Esses dados poderão auxiliar na implantação de ações para melhorar a qualidade de vida desses idosos.

## INTRODUCTION

Frailty in the elderly can be defined as a multifactorial syndrome that occurs due to a decrease in metabolic activities and reserves, difficulty in maintaining homeostasis, and vulnerability to stressors, leading to increased risk for disabilities.^1^

Advanced age is not a synonym of frailty and this is not present in all elderly, however, it can be affirmed that with the aging global population, a sharp increase in the prevalence of frailty can be expected.^2^

The risk factors and outcomes of frailty include falls, hospitalizations and mortality in frail elderly, which may occur in the presence of comorbidities^1,3,4^ or the absence of chronic diseases.^5^ The prevalence of frailty in elderly ranges from 5% to 58%.^3^

Recently, the main research group^6^ that studies the theme published a definition of physical frailty in elderly: "a medical syndrome with multiple causes and contributors that is characterized by diminished strength, endurance, and reduced physiologic function that increases an individual's vulnerability for developing increased dependency and/or death".

To evaluate frailty, the model most widely used is frailty as a phenotype measurable by five biological criteria: unintentional weight loss of more than 4.5 kilograms or 5% of body weight in the past year; fatigue, or exhaustion while doing regular activities; low hand-grip strength, which can indicate muscular weakness; decreased gait velocity, also reported as slowness when walking 4.6 meters on a flat surface; and low physical activity in comparison with one year ago, indicating low rate of energy expenditure.^1^

The inclusion of cognitive evaluation in frailty diagnosis has been discussed in some investigations, and in this sense there are studies that have included cognitive performance as a component to evaluate frailty.^3,7,8^

Cross-sectional studies demonstrate the link between frailty and cognitive performance^1^ and longitudinal studies show the relationship between frailty and the emergence of cognitive changes, cognitive impairment and dementia.^9,10^

The review conducted by Robertson, Savva and Kenny (2013), showing the relationship between frailty and decline in cognitive function, sought to establish the causal mechanisms and also found a relationship between worsening of cognitive domains and frailty. Perception peed, episodic memory, semantic memory and working memory have been associated with frailty. Commands, immediate memory, attention, verbal fluency and the clock drawing test are associated with worse performance in frail older adults. In the review, there were studies pointing to the existence and nonexistence of a relationship between memory and frailty.^11^

Drawing on this recent review of the data,^11^ the aim of the present study was to analyze the relationship between cognition and frailty in the elderly, focusing on studies conducted in low-middle income countries.

## METHODS

The present study comprised a systematic literature review, conducted based on previously established steps of search strategies, identification, screening, selection and eligibility of studies. Some criteria published on methods for the preparation of systematic reviews were adopted.^12^

The search for scientific articles took place between January and August 2014, using the LILACS, SCOPUS, SciELO, PsycINFO, PubMed and Web of Science databases. The descriptors for the search were obtained from MeSH and DeCS. The following operations on the databases were performed: (cognition AND frail elderly). Additional strategies with controlled and uncontrolled operators were employed: (cognition AND frailty AND elderly), (cognition AND frailty syndrome AND elderly), (cognition AND health vulnerability AND elderly).

The following inclusion criteria were used for article selection: publications in peer-reviewed journals published between January 2010 and August 2014, in English, Spanish or Portuguese. Reviews and meta-analysis articles were not included in the process of study selection. The search and inclusion of studies were performed independently and blindly by two evaluators (AGB and ESR), who evaluated the database compiled titles, abstracts or both, resolving discrepancies in consensus meetings.

The studies that met the following criteria were considered eligible:

(1) cross-sectional and longitudinal studies with elderly;
(2) studies that investigated the association between cognition and/or cognitive impairment (CI) - studies about dementia and Mild Cognitive Impairment (MCI) were included - with frailty and/or frailty criteria; and
(3) studies that evaluated cognition and frailty through validated methods in the literature.

Identification on databases was carried out using the search strategies outlined previously. Studies duplicated across databases were excluded. The studies to be included in the eligibility stage were selected by reading titles, abstracts or both. The eligibility criteria were then applied by critically reading the studies in full. Those studies that did not meet the criteria or did not address the research question were excluded.

## RESULTS

**Figure 1:**
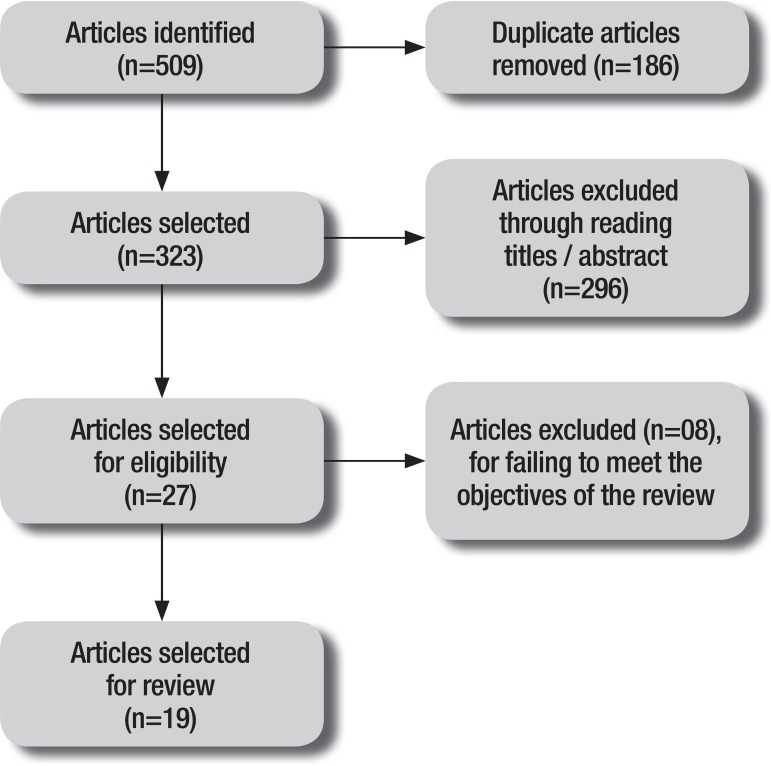
Summary of selection of articles for review.

A summary of the methods used and the findings is given in Figure 1. Of the number of articles initially identified in the database (n = 509), a total of 19 studies were selected for this review.

Of the total studies in the review, 10 (52.6%) had a cross-sectional methodology and 9 (47.4%) included follow-up of subjects. Two (10.5%) studies were published in 2010, 3 (15.8%) in 2011, 5 (26.4%) in 2012, 7 (36.8%) in 2013 and 2 (10.5%) were published in 2014. Most studies were carried out in Brazil (n=6, 31.6%), 4 (21%) in the United States, 2 (10.5%) Mexico, 2 (10.5%) Canada and 5.2% were published in each of the countries South Korea, Finland, China and Poland. Table 1 shows the main information for the cross-sectional studies while Table 2 shows the information for longitudinal studies.

**Table 1:** Cross-sectional studies on the relationship between cognition and frailty in elderly.

Study	Place	Demographics	Frailty measurement	Cognition measurement	Principal findings
Ávila-Funes et al. (2011)	Mexico City, Mexico	N=475 Age: 78.1 53.9% female	Fried et al. criteria	MMSE Isaacs Set Test	16% CI Frailty was associated with cognitive impairment, however without statistical significance (p=0.063)
Han, Lee, Kim (2013)	Twenty-five regions, South Korea	N=10388 Age: 65+ 58.9% female	Fried et al. criteria	MMSE	22.1% CI in non-frails 32.8% CI in pre-frails 55.8 % CI in frails 10% frail 46% pre-frail 44% non-frail in women Global cognition and cognitive domains associated with frailty
Kulmala et al. (2014)	Kuopio, Finland	N=654 Age: 82	Fried et al. criteria	MMSE	26% CI 14% frail 48% pre-frail 38% non-frail Association between cognitive impairment and frailty. Risk for cognitive impairment and dementia
Langlois et al. (2012)	Quebec, Canada	N=83 Age: 74.26 74.4% female	Fried et al criteria, Modified Physical Performance Test and Edmonton	MMSE, WAIS-III, Trail Making Test and Rey Auditory Verbal Learning Test	Mean MMSE non-frail = 28.06 (±1.46) Mean MMSE frail = 28.24 (±1.48) 47% frail 53% non-frail Differences in executives function and processing speed between the two groups.
Macuco et al. (2012)	São Paulo, Brazil	N=384 Age: 72.3 67.2% female	Fried et al. criteria	MMSE	21.2% CI 8% frail 54.2% pre-frail 37.8% non-frail Poor performance on cognitive domains in frail elderly
Moreira, Lourenço (2013)	Rio de Janeiro, Brazil	N=847 Age: 76.3 66.9% female	Fried et al. criteria	MMSE	Mean MMSE = 25.47 (±3.37) 9.1% frail 47.3% pre-frail 43.6% non-frail Frail elderly had worse cognitive performance
Neri et al. (2013)	Seven cities, Brazil	N=3478 Age: 72.9 67.7% female	Fried et al. criteria	MMSE	24.8% CI 9.1% frail 51.8% pre-frail 39.1% non-frail. Association between frailty and cognition
Sánchez-García et al. (2014)	Mexico City, Mexico	N=1933 Age: 70.1 58% female	Fried & Walston criteria	MMSE	17.4% CI 15.7% frail 33.3% pre-frail 51% non-frail Cognitive impairment associated with pre-frailty
Santos et al. (2013)	Belem, Ermelino Matarazzo, Brazil	N=878 Age: 72.1 67.7% female	Fried et al. criteria	MMSE	Mean MMSE = 24.97\ 8% frail 50% pre-frail 42% non-frail Frail elderly had worse cognitive performance
Yassuda et al. (2012)	Ermelino Matarazzo, Brazil	N=384 Age: 72.3 67.2% female	Fried et al. criteria	Brief Cognitive Screening Battery and MMSE	16.6% CI 7% frail 54.2% pre-frail 38.8% non-frail Association between cognition and frailty

MMSE: Mini-Mental State Examination; CI: cognitive impairment.

**Table 2:** Longitudinal studies on the relationship between cognition and frailty in elderly.

Study	Place	Demographics	Frailty measurement	Cognition measurement	Principal findings
Alencar et al. (2013)	Belo Horizonte, Brazil	N=207 Age:78.5 76.8% female	Fried et al. criteria	MMSE and CDR	6.4% CI in non-frails 25% CI in pre-frails 58.3% CI in frails 23.2% frail 54.1% pre-frail 22.7% non-frail Association between frailty and MMSE
Auyeung et al. (2011)	Hong Kong, China	N=2737 Age: 71.6 73.1% female	Five components*	MMSE	Mean MMSE in men = 27.4(±2.25) Mean MMSE in women = 25.8(±2.80) Frailty components can predict cognitive impairment.
Boyle et al. (2010)	Chicago, USA	N=750 Age: 79 76.4% female	Four components**	MMSE + twenty tests	Mean MMSE = 28.4 (±1.7) Frailty associated with decline in five cognitive domains (episodic memory, semantic memory, working memory, perception speed, and visuospatial abilities).
Cano et al. (2012)	Five states, USA	N=1815 Age:73.3 62.4% female	Fried et al. criteria	MMSE	CI in 8.8% of living group CI in 10.% of deceased group Association between cognitive impairment and frailty
Gray et al. (2013)	Seattle, USA	N=2619 Age:76.8 60% female	Fried et al. criteria	Ten tests	19.8% dementia incidence 8.1% frail 52.9% pre-frail 39% non-frail Interaction between cognition and frailty in elderly with dementia
Jacobs et al. (2011)	Jerusalem, Israel	N=840 Age: 85-90 53.3% female	Fried et al. criteria	MMSE	Mean MMSE = 25.6(±5.4) 19.5% frail 56% pre-frail 24.5% non-frail Association between cognitive impairment and frailty
Matusik et al. (2012)	Krakow, Poland	N=86 Age: 83.8 76.7% female	CSHA-CFS	MMSE	55.8% severe CI 75.6% frail Severe frailty and cognitive impairment can predict mortality
Raji et al. (2010)	Five states, USA	N=942 Age: 73.3 57.8% female	Fried & Walston criteria	MMSE	Mean MMSE of <21 group = 18.6(±2.4) Mean MMSE of ≥21 group= 26.1(±3.2) Worse cognition had association with risk of frailty Increased slowness in cognitive impairment
Rolfson et al. (2013)	Alberta, Canada	N=164 Age: 74 53% female	Edmonton and Fried et al.	MMSE, PCT, LCT	Mean MMSE = 26.7 (±4.32) Association between frailty and neurocognitive speed

MMSE: Mini-Mental State Examination, CDR: Clinical Dementia Rating, PCT: pattern comparison test, LCT: letter comparison test, CSHA-CFS: Canadian Study of Health and Aging-Clinical Frailty Scale. CI: cognitive impairment.

* appendicular skeletal muscle mass (ASM), hand-grip strength, timed chair-stand test, walking speed, step length.

** slowness, weakness, exhaustion, body composition.

Of the cross-sectional studies, 90% categorized the elderly according to frailty levels, whereas 8 (80%) used the frailty evaluation proposed by Fried et al. (2001),1 and all included the Mini-Mental State Examination (MMSE) of Folstein, Folstein and McHugh (1975)13 in their cognitive evaluation. Except for the Ávila-Funes et al. (2011)14 study, the association between cognitive alterations and frailty was statistically proven.

Of the longitudinal studies, 44.4% categorized the elderly according to frailty levels, 4 used the frailty evaluation proposed by Fried et al. (2001)^1^ and 8 studies included the MMSE in their cognition evaluation. Of the 9 longitudinal studies, 8 were conducted outside Latin America.

Out of all the studies reviewed, 10 (52.6%) reported specific results of an association between frailty components and cognitive domains. Among these studies, 50% found slowness, 40% muscular weakness, 20% exhaustion and 10% linked weight loss, low chair stand speed and step length with cognitive impairments. Regarding cognitive domains, memory subtypes (episodic, semantic, working, storage, encoding and immediate) were most strongly associated with frailty (30%), followed by processing/perception speed (20%), temporal orientation (20%) and visuospatial skills (20%). The executive functions, verbal fluency, attention, commands, language and judgment were associated in 10% of these studies.

One study reviewed found an association between frailty and the diagnosis of MCI, two highlighted risk of dementia in frail elderly, with the greatest risk being for non-Alzheimer's Disease (AD) dementia, and three found increased prevalence of mortality in frail elderly with cognitive impairment.

**Cross-sectional studies.** Some studies established a direct relationship between cognitive impairment and frailty in elderly. The study of Ávila-Funes et al. (2011) in the City of Mexico involving a sample of 475 participants with a mean age of 78.1 years, 46.1% men and 49.5% married, found a 16% prevalence of cognitive impairment. Frailty was associated with cognitive impairment, however without statistical significance (p=0.063). The results showed that before the frailty components are adjusted, except for weight loss, all were associated with activities of daily living. It was concluded that cognitive impairment and low physical activity are the main contributing factors to frailty.^14^

Also in Mexico, 1933 seniors evaluated by Sánches-Garcia et al. (2014) had a prevalence of 15.7% frailty, 33.3% pre-frailty and 51% non-frailty. The majority (58%) of the elderly were women, with a mean age of 70.1 years. A total of 17.4% had cognitive impairment and 22.7% showed depression symptoms. Cognitive impairment was associated with both frailty and pre-frailty in the elderly assessed.^15^

Brazilian studies have shown similar results concerning sociodemographic characteristics, frailty prevalence and cognitive impairment. Moreira and Lourenço (2013) conducted a study in Rio de Janeiro city with a random sample of 847 seniors. Participants were predominantly women (66.9%), with a mean age of 76.6 years, 62.6% Caucasian and 44.1% married. The prevalence of frailty was 9.1%, while 47.3% of participants were pre-frail and 43.6% non-frail. Mean MMSE score was 25.47 (±3.37). Multivariate logistic regression showed a strong association between cognition and frailty. The frail elderly, predominantly widowed women, had worse cognitive performance.^16^ Another Brazilian study with 3478 community elders showed very similar results. In the multicentric study by Neri et al. (2013), the majority were women (67.7%), married (48%) or widowed (36.4%), living with their child's family (52.6%), householders (64.5%) with 1 to 4 years of formal education (49%), 28.8% illiteracy and 24.8% cognitive impairment. Of the sample, 9.1% were frail, 51.8% pre-frail, 39.1% non-frail, and 25.4% had cognitive impairment. Among the frail elderly, there was a higher proportion of women and individuals who were aged 80 years or older, widowed, illiterate and with cognitive impairment.^17^

The study published by Santos et al. (2013) evaluated 878 elderly without cognitive impairment. The age range was 65-92 years, the majority 594 (67.7%) female, of mixed race - "mulattoes, caboclos" and brown - (50.9%), married (44.5%), or widowed (35.1%). Regarding frailty, 42% were non-frail, 50% pre-frail and 8% frail, with an average MSSE score of 24.97 points. The MMSE results showed statistically significant differences between frail, pre-frail and non-frail elders, and the regression analysis for the MMSE showed that frail elderly differed from non-frail whereas the same disparity did not occur between pre-frail and non-frail groups.^18^

Cognitive domains and specific frailty criteria were present in the following studies. In Canada, Langlois et al. (2012) compared frail and non-frail elderly for physical, cognitive and psychological dimensions. A total of 39 frail and 44 non-frail seniors were compared on various physical activity, cognition and quality-of-life measures. The mean MMSE score in non-frails was 28.06 (±1.46) and frails was 28.24 (±1.48). The study showed differences between the groups regarding executive functions and processing speed, with frail individuals having the worst performance. Furthermore, frail elderly reported lower self-perceived physical ability, cognition, affectivity, housekeeping efficacy and physical health.^19^ Han, Lee, Kim (2013) found, in a study of 10338 elderly from South Korean communities, that all cognitive domains were inversely correlated with the risk of frailty. The results also showed that 58.9% of the sample consisted of women, 9.3% were frail, 42.3% pre-frail and 48.4% non-frail. The frailty prevalence was higher among women (10%) compared to men (8.3%), where 37.1% of men and 46% of women were pre-frail and 54.7% of men and 43.9% of women non-frail. The prevalence of CI was 22.1% in non-frails, 32.8% in pre-frails and 55.8 % in frails. Cognitive impairments were associated with high levels of frailty and risk of frailty. High scores for temporal, register, attention and judgment orientations were associated with a lower risk of frailty in both sexes, while in women, memory, language and visuospatial ability were also associated with low probability of frailty.^20^

In a poor Brazilian community, Yassuda et al. (2012) analyzed 384 older adults using frailty measures and cognition as evaluated by the Brief Cognitive Assessment Tool (memorization of 10 black and white pictures, animal, verbal fluency category and the Clock Drawing Test) and by the MMSE. The prevalence of MMSE impairment was 16.6% for the overall sample, 11.6% for non-frails, 16.8% for pre-frails and 42.8% for frails. The frail elderly had the worst cognitive performance when compared to pre and non-frail elderly. Muscular strength was associated with MMSE performance, while the slowness frailty criteria was associated with verbal fluency and the Clock Drawing Test.^21^ Macuco et al. (2012), based on the same sample, found a prevalence of frailty of 8%. Of the participants, 54.2% were classified as pre-frail and 37.8% as non-frail. Regarding cognition, 21.2% had cognitive impairment, whereas 15.6% of non-frail, 22.3% of pre-frail and 38.7% of the frail scored below the cut-off on the MMSE. The frailest individuals had the worst performance on the MMSE. Being considered frail was associated with worse performance in temporal orientation, immediate memory and command difficulty.^22^

A Finnish study proposed that a risk of more severe cognitive impairment, resulting in dementia diagnosis was associated with frailty level. Kulmala et al. (2014) in a study of 654 older adults found a 14% frailty prevalence. Of the elderly assessed, 171 (26%) had cognitive deficits and 134 (21%) were diagnosed with dementia. The cognitive impairment prevalence in frail elderly was 64%. Frailty was associated with cognitive impairment. The regression analysis showed that frail elderly were eight times more likely to develop cognitive impairment, six time more likely to develop vascular dementia and four times more likely to develop dementia due to AD.^23^

**Longitudinal studies.** The longitudinal studies, beyond the cognition and frailty relationship, revealed that components of frailty were associated with poorer performance in cognitive domains, as well as showing frailty risk for cognitive impairment, onset of dementia and the risk of mortality during the follow-up of participants.

The Canadian study of Rolfson et al. (2013) evaluated the interaction between neurocognitive speed and operationalized frailty in two different ways. A sample of 164 participants without dementia were followed annually for three years. Besides the evaluation by MMSE, neurocognitive speed was defined by other tests. Frailty was assessed using the Edmonton Frail Scale and the criteria of Fried et al. (2001).^1^ Regression analysis showed that both evaluations of frailty were associated with low neurocognitive velocity, however, only the evaluation criteria by Fried et al. (2001)^1^ was associated with the cognitive assessment by MMSE. Another important result was that, while monitoring the sample, neurocognitive speed decreased with increased frailty when evaluated by the criteria of phenotype frailty.^24^

In China, Auyeung et al. (2011) followed a sample of 2737 cognitively normal community-dwelling elderly. Frailty was measured by the following aspects: decrease in appendicular skeletal muscle mass, reduced grip strength, reduced speed in rising from a chair, weight loss, slow gait and shorter step length. At baseline, the mean MMSE score in men was 27.4(±2.25) and in women was 25.8(±2.80) points. Results indicated that in men, all frailty measures were significantly associated with MMSE performance, and decreased their total score during the four-year follow-up. After adjusting for age, years of formal education and MMSE score, appendicular muscle mass and walking speed were proven insignificant. In women, all frailty measures, except for appendicular muscle mass and weight loss, were significantly associated with MMSE. Furthermore, weaker grip strength remained significant after adjusting for age, years of formal education and MMSE performance.^25^

A follow-up study conducted in Brazil was also included. Alencar et al. (2013) evaluated the association between frailty and cognitive impairment and the incidence of cognitive impairment in a sample of 207 elderly, with or without cognitive impairment, followed for 12 months. Cognition was evaluated by the MMSE and the Clinical Dementia Rating (CDR) scale. In the first evaluation, 76.8% were women, mean age was 78.5 years, and 47 (22.7%) participants were classified as non-frail, 112 (54.1%) pre-frail and 48 (23.2%) were classified as frail. Around 6.4% of non-frail, 25% of pre-frail and 58.3% of frail had cognitive impairment. Of the initial 207 participants, 187 were reevaluated (12% lost to follow-up). Frailty was associated with subsequent decline in cognitive function and cognitive impairment on the CDR. In the study, there was no relationship between frailty and the incidence of cognitive decline, however the proportion of new cases of cognitive impairment was 4.9% among non-frail, 8.9% in pre-frail and 13.3% in frail.^26^

Raji et al. (2010), in a study with a 10-year follow-up, examined whether poor cognition could predict frailty risk in non-frail elderly from five American states (Texas, New Mexico, California, Colorado and Arizona). A total of 942 non-frail elderly were interviewed, 57.8% of whom were women, with a mean age of 73.3 years. A modified version of the Fried and Walston evaluation of frailty was used. The version encompassed four items, including involuntary weight loss, exhaustion, fatigue and weakness. The sample was divided into two groups (MMSE<21 and MMSE≥21). In general, estimation equation models testing the relationship between MMSE and risk of becoming frail over a period of 10 years showed significance. This association persisted regardless of age, sex, marital status, education, time and medical conditions, indicating that non-frail elderly with low cognition had a 9% higher chance per year of becoming frail, in comparison to the individuals with good cognition. An important finding was that from the first follow-up to the second, 30.9% of the elderly with cognitive changes and 26.3% of the elderly without cognitive impairment fulfilled the criteria for weight loss. From the second follow-up to the last, slowness had increased 25% in the cognitive changes group versus 18.1% in the non-impaired group.^27^

Frailty was shown as a risk factor for Mild Cognitive Impairment (MCI) by Boyle et al. (2010), that followed 750 elderly without baseline cognitive impairment for 12 years. Frailty was evaluated according to four criteria: grip strength, walking speed, body composition and fatigue. The greater the impairment on these criteria, the frailer the elder. Cognition was more widely evaluated by the MMSE and other tests. Regarding the sample, mean age was 79 years, formal education 14.5 years and 76.4% were women. The men were less frail than the women and frailty was inversely associated with global cognitive performance. During the follow-up, 40% of participants developed MCI, and the presence of each physical frailty component was associated with a faster rate of global cognitive decline and with the five evaluated domains (episodic memory, semantic memory, working memory, perception speed and visuospatial abilities). Furthermore, results also showed that lower grip strength and walking speed were associated with the risk of first occurrence of MCI.^28^

Risk for low occurrence of dementia influenced by frailty was shown in Mexico by Gray et al. (2013). The study sample comprised 2619 participants aged ≥ 65 years, without dementia at study baseline. Frailty was measured according to criteria by Fried et al. (2001),^1^ and cognition was assessed based on ten neuropsychological tests. Of the sample, 8.1% were frail and 39% non-frail. For other causes of dementia, interaction was found between cognitive score and frailty. Over a mean follow-up of 6.5 years, 521 (19.8%) participants developed dementia (of which 448 developed AD). In the age, gender, education and race-adjusted model, the risk of frailty rate was 1.78. In the fully adjusted models, frailty risk rate was 1.20 for all-cause dementia, 1.08 for AD and 2.57 for non-AD. Frailty was associated with higher risk of developing non-Alzheimer's dementia. Slow walking speed was associated with the risk of non-Alzheimer's dementia. However, the regression analysis showed that muscular weakness and exhaustion represented significant dementia risks.^29^

Cognitive impairment and frailty, independently or otherwise, were associated with risk of mortality among elderly in three studies. Jacobs et al. (2011) followed 840 community elders in Jerusalem, Israel, for 5 years. Of the sample, 19.5% were frail, 56% pre-frail and 24.5% non-frail, with 53.3%, 15% and 7.4% of these groups scoring ≤ 24 on the MMSE, respectively. Among frail, pre-frail and non-frail, the mortality rate in 5 years was 44.5%, 20.4% and 13.6%, respectively. Mortality among frail individuals with cognitive changes was 54.2%, and without changes was 54.9%. Frailty was significantly associated with cognitive impairment and was predictive of subsequent mortality.^30^

Matusik et al. (2012) followed 66 women and 20 men residing at nursing homes in Poland. Frailty prevalence was 75.6%, where 34.9% had severe frailty, 23% moderate frailty and 17.4% mild frailty. Severe cognitive impairment was present in 55.8% of the elderly. The residents with severe frailty and severe cognitive impairment accounted for 33.7% of the sample and 50% of the deceased within 12 months. The follow-up showed that the one-year mortality was higher in those with severe frailty and severe cognitive impairment compared with other residents. The authors concluded that frailty, dementia and cognitive impairment were predictors of higher mortality rate in institutionalized seniors.^31^

Finally, in North America, Cano et al. (2012) examined the association between frailty and cognitive changes as mortality predictors within a 10-year period in a sample of 1815 Mexican-American elderly. By the end of the follow-up, 690 cases of death of participants in the initial sample were confirmed. Among the surviving seniors (n=917), the mean age was 73.3 years and 62.4% were women. Out of the sample, 8.3% of the living group and 10.2% of the deceased group had cognitive impairment. MMSE scores decreased over time and the percentage of frail individuals increased linearly. Frailty and cognitive changes were associated. However, frailty and cognitive impairment were independent risk factors after controlling for the others variables. The results showed that frailty was a higher risk factor for mortality than cognitive impairment, where mortality rate was higher in frail elderly than on pre-frail or non-frail groups. Individuals who were male, older, married, with hip injuries and frail elderly were significantly more likely to be in the deceased group during the follow-up.^32^

Some peculiarities are evident, especially in cross-sectional studies. The prevalence of frailty in Latin American studies of the elderly was around 10% and cognitive impairment ranged from 16% to 25% in the samples. Mean scores on the MMSE were around 25 points while in other countries the average points on the test is greater. In these other countries, the prevalence of cognitive impairment was from 20% to 55%. The prevalence of frailty was similar across all studies.

Lastly, several factors may contribute to higher frailty levels and consequently worse cognitive performance. Among them are advanced age, low education, low financial income, female gender, widowed/unmarried, low body weight and poor nutritional status, dependence in functional and daily living activities, symptoms of depression, presence of comorbidities, worse perceived health, use of medications and drugs and the use of health services.^14-32^

## DISCUSSION

**Figure 2:**
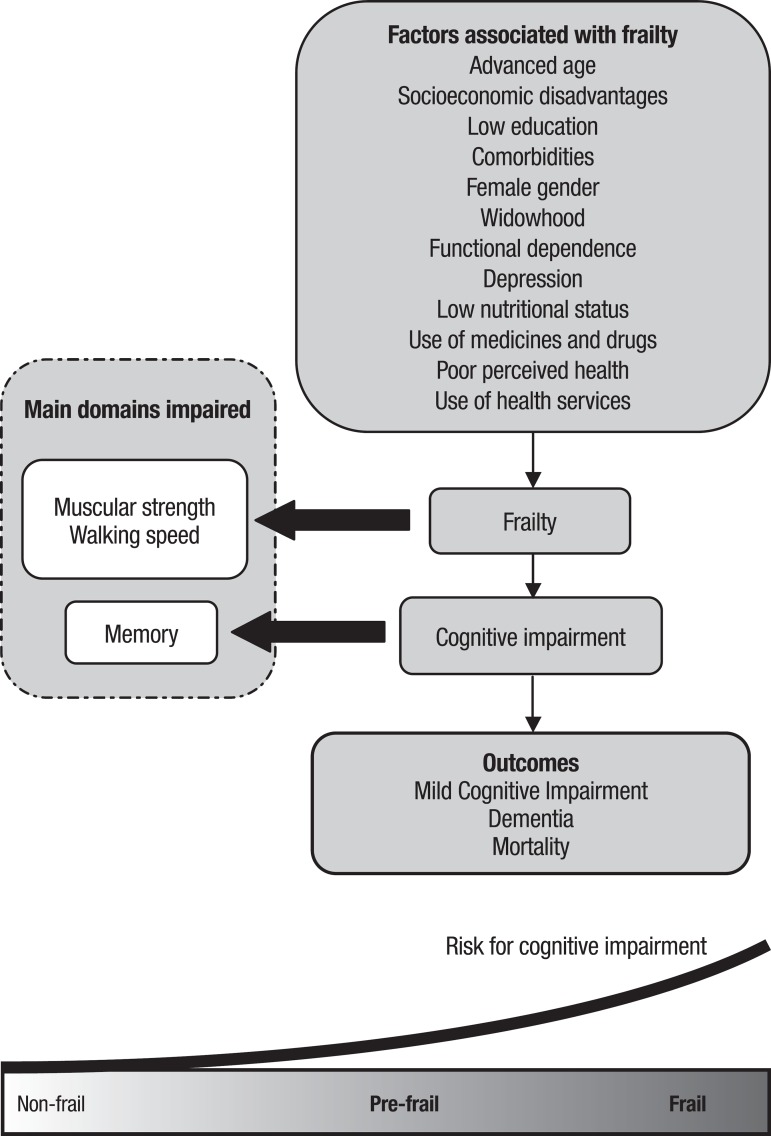
Model of association between factors, frailty, cognitive impairment and their outcomes in older adults.

According to the results of the reviewed studies, a model, illustrated in Figure 2, was produced in order to better understand the frailty-associated factors. Frailty is a condition or outcome influenced by several factors elucidated by the reviewed studies. In the scope of frailty, muscular strength and walking speed are impaired, as is cognitive performance, with memory being the most impaired. Among the major outcomes of this are MCI, dementia and the greater risk of mortality in the elderly. Figure 2 illustrates the increased risk for cognitive impairment showing that its severity may be influenced by higher levels of frailty in the elderly.

Another review on evidence and causal mechanisms reported that frailty can increase the risk of future cognitive impairment. An integrated cycle of frailty, cognitive impairment and mental health was constructed, where some of the associated factors identified in this review are present in the cycle, such as depression, dependency, comorbidities (diseases) socioeconomic disadvantages (low social engagement) and low nutritional status (chronic undernutrition).^11^

Also, the same review highlighted the need for studies determining which measures of frailty could be used to best identify the risk of cognitive decline in aging.^11^ The systematic review of Sternberg et al. (2011) emphasized the importance of inclusion of disability, cognition and mood in frailty elements.^33^ An overview of Bouillon et al. (2013) reported that the reliability and validity of frailty measures are rarely examined, but the Fried et al. criteria is the most frequently used,^34^ a situation also observed in this review. Furthermore, a Korean study reported that the Fried et al. criteria is better than other types of evaluation, but a new frailty index called KFI (KLoSHA Frailty Index) was an excellent frailty measure, correlated with hospitalization and able to predict subsequent functional decline.^35^ However, a good measure that evaluates frailty and its risk for cognitive impairment in elderly is required.

Based on this systematic review of the literature, drawing on studies from a large number of databases, it can be concluded that cognition is associated with frailty, especially when operationalized as a physical syndrome marked by vulnerability to stressors in the elderly.

Frailty components, particularly slowness and muscular weakness, are associated with cognitive functioning where memory seems to be the most affected cognitive function. Cognitive impairment is more prevalent in frail elderly, and the greater the frailty, the higher the risk for MCI and dementia. The concomitant presence of frailty and cognitive changes can strongly predict mortality in the elderly.

This review explored a substantial number of studies from Brazil and Latin America, mostly cross-sectional investigations. Strengths of the study include the sample characteristics of low educational level and poor financial support and the relationship of these factors with cognitive function and frailty in this group. In theory, the elderly in the poorest communities are more vulnerable than elderly from wealthier communities, but longitudinal studies should be conducted in developing countries.

The scarcity of studies reviewing the subject represents a study limitation regarding the discussion of results. It is hoped that the present results prompt further research on the relationship between physical and mental health and the aging process, and that this serves as input for the creation, evaluation and discussion of actions involving attention and care in Gerontology.
